# The Minimal Clinically Important Difference for Achievement of Substantial Reperfusion With Endovascular Thrombectomy Devices in Acute Ischemic Stroke Treatment

**DOI:** 10.3389/fneur.2020.524220

**Published:** 2020-10-05

**Authors:** Chun-Jen Lin, Jeffrey L. Saver

**Affiliations:** ^1^Neurological Institute, Taipei Veterans General Hospital and School of Medicine, National Yang-Ming University, Taipei, Taiwan; ^2^Comprehensive Stroke Center and Department of Neurology, David Geffen School of Medicine, University of California, Los Angeles, Los Angeles, CA, United States

**Keywords:** thrombectomy, MCID (minimal clinically important differences), ischemic stroke, device, technical efficacy

## Abstract

**Background and Purpose:** Recent noninferiority clinical trials of novel endovascular thrombectomy devices for acute ischemic stroke have used substantial reperfusion as the primary outcome of achievement. Determining the minimal clinically important difference (MCID) is an essential step for the design of noninferiority clinical trials.

**Materials and Methods:** We surveyed international neuro-interventionalist and noninterventional vascular neurologist investigators. The questionnaire included demographic characteristics, level of clinical experience, and their MCID clinical scenario-based judgment regarding the MCID for the outcome substantial reperfusion (thrombolysis in cerebral infarction score 2b-3) within 3 passes.

**Results:** Survey responses were received from 58 of 200 experts. Among responders, 75.9% were neuro-interventionalists (most commonly interventional neuroradiologists and interventional neurologists, followed by endovascular neurosurgeons), and 24.1% were noninterventional vascular neurologists; 87.9% had been in practice for more than 5 years, and 67.3% devoted more than half of their practice to stroke care. Responder–nonresponder and continuum of resistance analysis indicated responders were representative of the full expert population. Among experts, the median MCID for substantial reperfusion was 3.1–5% (interquartile range 1.1–3% to 5.1–10%). MCID distributions did not differ among neuro-interventionalists and noninterventional vascular neurologists.

**Conclusions:** Neuro-interventionl and noninterventional stroke experts judged that the minimal clinically important difference in comparing thrombectomy devices for achieving substantial reperfusion is 3.1–5%. This MCID, lower than noninferiority margins used in several recent clinical trials, can inform trial designs and clinical development.

The appropriate clinical trial designs to assess device treatments for a disease may be divided into four broad types, based on 2 types of endpoint and 2 types of design. The two types of primary endpoints are (1) technical efficacy endpoints, used when the trial is assessing the device's effectiveness as a tool (does it do what it says?), and (2) clinical efficacy endpoints when the trial is assessing the device's effectiveness as a care strategy (does the patient have a better final health state as a result?). The two types of designs are (1) superiority clinical trials, in which a novel device is tested to determine if it yields better technical or clinical outcomes than a standard device or medical therapy alone, and (2) noninferiority clinical trials, in which a novel device is tested to determine if it yields technical or clinical outcomes at least as good as a standard device or medical therapy alone.

Trials using technical, rather than clinical, efficacy outcomes may be of particular importance at early and at final stages in the initial development of a device class and use case. Early in device development, iterative innovation in device design to optimize its technical performance is a leading concern. Once device designs are sufficiently mature, it is desirable to perform superiority trials against older care strategies not using the device class to demonstrate that using the device class does ultimately improve the final clinical outcomes of patients. When a device class has been shown to have clinical utility, further novel, within-class devices may often be adequately assessed again with the use of technical efficacy primary endpoints. At this stage, when the device class has already attained a generally high level of technical performance success, noninferiority trials with technical efficacy endpoints are useful, in which novel device variations are shown to be at least as good as existing, approved devices.

The development of endovascular thrombectomy (EVT) devices for acute ischemic stroke due to large vessel occlusion (AIS-LVO) has recently generally followed this trajectory. A wave of several superiority design trials were first performed and demonstrated the superiority of endovascular thrombectomy over medical therapy alone (1–9). Thereafter, trials assessing successor variations in thrombectomy device design have generally used noninferiority designs, seeking to demonstrate that newer devices are at least as good as existing devices (10–12).

For noninferiority clinical trial design, even more so than for superiority clinical trial design, determining the minimal clinically important difference (MCID) in an outcome is a crucial, and challenging, step that affects trial power. The MCID is the smallest change in a disease outcome that a patient and/or a care provider would identify as worthwhile (13). For superiority trials, seeking to determine if new treatment A is better than conventional treatment B, the MCID establishes the threshold difference at which superiority can be declared. If treatment A yields an incremental increase in favorable outcomes that exceeds the MCID, then superiority is established. For noninferiority and equivalence trials, the MCID establishes the threshold value (“margin”) at which noninferiority or equivalence can be declared. If treatment A does not yield an incremental increase in favorable outcomes that exceeds the MCID, then noninferiority is established. For both superiority and noninferiority/equivalence trials, requisite sample size is inversely related to the amplitude of the MCID. The smaller the MCID for a particular outcome, the larger a trial must be in order to be adequately powered to determine if a novel treatment exceeds or does not exceed that threshold.

However, for EVT treatment of AIS-LVO, the MCID has not been formally established for the most commonly employed technical efficacy outcome: achieving substantial reperfusion (i.e., achieving a thrombolysis in cerebral infarction, TICI, scale score of 2b-3). During the conduct of a recent noninferiority meta-analysis of randomized trials comparing different EVT techniques, a systematic literature search identified two expert survey studies characterizing the MCID for the clinical efficacy outcome of functional independence (modified Rankin Scale 0–2) at 3 months but no expert survey study characterizing the MCID for the technical efficacy outcome of achievement of substantial reperfusion (14). The current study was, therefore, undertaken to provide that desirable additional frame.

Approaches to establishing the MCID for a particular outcome are of 3 broad types: Delphi expert–based approaches, anchor-based approaches, and distribution-based approaches (15). For an acute-onset disease such as stroke, the Delphi expert–based approach is generally preferred (16), especially for a technical efficacy outcome such as reperfusion, which requires technical expertise to appreciate, rather than patient-level subjective experience of a disease state. Therefore, we undertook a Delphi-expert survey study of leading international neuro-interventionalists and noninternventional vascular neurologists to the MCID of achieving substantial reperfusion with endovascular thrombectomy devices in patients with AIS-LVO.

## Materials and Methods

### Participants

To identify a large pool of potential survey recipients who were neuro-interventionalists or noninterventional vascular neurologists with expertise in modern endovascular thrombectomy care, we examined the study center listings in 15 recent large, multicenter endovascular thrombectomy studies, including the eight randomized superiority-design trials of endovascular mechanical thrombectomy vs. medical therapy alone identified in a recent systematic review (MR CLEAN, ESCAPE, SWIFT-PRIME, EXTEND-IA, REVASCAT, THRACE, PISTE, THERAPY) (17), one superiority design trial comparing contact aspiration vs. stent retriever (ASTER) as well as the four controlled noninferiority design trials comparing different endovascular mechanical thrombectomy techniques identified in another recent systematic review (SWIFT, TREVO 2, Penumbra-3D, ARISE II) (14), and three recent large multicenter observational studies in the United States (TRACK, STRATIS, NASA). For each study, we identified the 10 highest enrolling centers and, for those centers, abstracted the names of (1) the site principal investigator, (2) the site co-investigator whose last name was earliest in alphabetic order, and (3) the site co-investigator whose last name was last in alphabetic order. We sought email addresses for all these individuals from public and specialty society sources, including hospital websites, PubMed, and LinkedIn. Those without searchable emails were excluded.

### Survey Design

The survey consisted of five questions on a single web page. The first four questions elicited information regarding responders' specialty, academic appointment level, years in clinical practice, and proportion of practice devoted to stroke care.

The fifth question addressed the MCID using the scenario and response options shown below:

Please imagine that a 70-year-old patient presents with an M1 MCA occlusion within 4 h of last known well and you have available two different thrombectomy devices, device A and device B. Based on large clinical trials, the devices have different rates of success in achieving substantial reperfusion (TICI 2b-3) within 3 passes. In how many additional patients, per every 1,000 treated, would device A, compared with device B, need to yield substantial reperfusion for you to consider device A to have a clinically worthwhile reperfusion advantage over device B?

a. *1–5*
b. *6–10*
c. *11–30*
d. *31–50*
e. *51–100*
f. >*100*

### Survey Process

The survey was distributed by using an Internet survey platform (SurveyMonkey Inc., San Mateo, California, USA, www.surveymonkey.com). Each individual received an initial invitation to participate by email, followed by 2 subsequent email reminders sent 1 week apart to those not initially responding.

### Statistical Analysis

To evaluate the representativeness of responders compared to nonresponders, two analytic approaches were undertaken. For demographic data characterizing physicians that were publicly available (sex and geographic location), we compared responders and nonresponders bivariately. For professional practice data that were not publicly available (specialty, academic appointment level, years in practice, and proportion of practice stroke-related) and for MCID responses as well, we used the continuum of resistance model (16, 18). We defined individuals who responded to the first email invitation as early responders and individuals who responded to the second or third invitation as late responders. Those with no response after all three invitation rounds were defined as nonresponders. Because late responders would have been categorized as nonresponders if the study had been stopped earlier, they are expected to act more like the nonresponders than the early responders in the spectrum of response (from always respond to never respond). Therefore, comparing the characteristics between early and late responders provides an estimate of the potential response bias. We used Statistical Package for the Social Sciences software (SPSS, IBM Corp. Released 2016. IBM SPSS Statistics for Windows, Version 24.0. Armonk, NY, USA) for the statistical analyses. Chi-square was used for categorical variables comparison between groups. A *p*-value < 0.05 was considered significant.

This study was determined by the UCLA institutional review board to be exempt from ethical approval and not require participant informed consent as it consisted only of survey procedures, and analysis was confined to deidentified data.

## Results

### Expert Identification and Response

The protocol for harvesting expert names from published trials and multicenter studies yielded 203 individuals, of whom 200 had active email addresses. Survey responses were received from 58 of 200 (29%).

### Demographic Characteristics

The characteristics of the survey participants are shown in Table 1. Three quarters of respondents were neuro-interventionalists, most commonly interventional neuroradiologists and interventional neurologists, followed by endovascular neurosurgeons. One quarter were noninterventional vascular neurologists. Nearly two thirds were from North America and one third from Europe with a small proportion from the Asia-Pacific region. Nearly half were senior faculty, and more than one quarter were midcareer faculty. Nearly 90% had been in practice for more than 5 years, and more than 60% had been in practice for more than 10 years. Nearly two thirds devoted more than half of their practice to stroke care.

**Table 1:** Characteristics of physician survey participants.

**Characteristic**	**Number (%), *n* = 58**
**Sex, Male**	56 (96.6)
**Geographic Location**	
North America	36 (62.1)
Europe	20 (34.5)
Asia-Pacific	2 (3.5)
**Specialties**	
Interventional Neuroradiology	20 (34.5)
Endovascular Neurosurgeon	6 (10.3)
Interventional Neurology	18 (31.0)
Non-Interventional Vascular Neurology	14 (24.1)
**Current Appointment Level**	
Clinical Instructor	4 (6.9)
Junior Faculty	10 (17.2)
Midcareer	17 (29.3)
Senior Faculty	27 (46.5)
**Years of Practice**	
0–5	7 (12.1)
6–10	15 (25.9)
11–15	13 (22.4)
>15	23 (39.7)
**Proportion of Practice Devoted to Stroke**	
<10%	0 (0)
10–50%	19 (32.8)
51–90%	20 (34.5)
91–100%	19 (32.8)

### Representativeness

Survey responders appeared representative of the entire solicited sample with no significant differences in geographic region or sex (Table 2).

**Table 2:** Evaluation for responder bias.

**Direct Comparison of Responders and Nonresponders, Number (%)**			
**Characteristic**	**Responders**	**Nonresponders**	***p*****-values**
	**(*****n*** **=** **58)**	**(*****n*** **=** **142)**	
**Sex, Male**	56 (96.6)	128 (90.1)	0.16
**Geographic Location**			0.49
America	36 (62.1)	76 (53.2)	
Europe	20 (34.6)	59 (41.3)	
Asia-Pacific	2 (3.5)	8 (5.7)	
**Continuum of Resistance Model Comparison of Early vs. Late Responders Number (%)**,			
**Characteristic**	**Early Responders**	**Late Responders**	***p*****-values**
	**(*****n*** **=** **42)**	**(*****n*****=** **16)**	
**Specialties**			0.94
Interventional Neuroradiology	14 (33.3)	6 (37.5)	
Endovascular Neurosurgeon	5 (11.9)	1 (6.3)	
Interventional Neurology	13 (31.0)	5 (31.3)	
Non-Interventional Vascular Neurology	10 (23.8)	4 (25)	
**Current Appointment Level**			0.94
Clinical Instructor	3 (7.1)	1 (6.3)	
Junior Faculty	8 (19.1)	2 (12.5)	
Midcareer	12 (28.6)	5 (31.25)	
Senior Faculty	19 (45.2)	8 (50)	
**Years of Practice**			0.09
0–5	7 (16.7)	0 (0)	
6–10	11 (26.2)	4 (25)	
11–15	11 (26.2)	2 (12.5)	
>15	13 (31.0)	10 (62.5)	
**Proportion of Practice Devoted to Stroke**			0.89
<10%	0 (0)	0 (0)	
10–50%	14 (33.3)	5 (31.3)	
51–90%	15 (35.7)	6 (25)	
91–100%	13 (31.0)	5 (31.3)	

Among the responders, a little less than three quarters of individuals were early responders and a little more than one quarter were late responders. When comparing early vs. late responders, there was no significant difference in the distribution of specialties, seniority, years of practice, or proportion of practice devoted to stroke care although a trend was noted for late responders to have been in practice longer than early responders (Table 2).

### Minimal Clinically Important Difference

**Figure 1:**
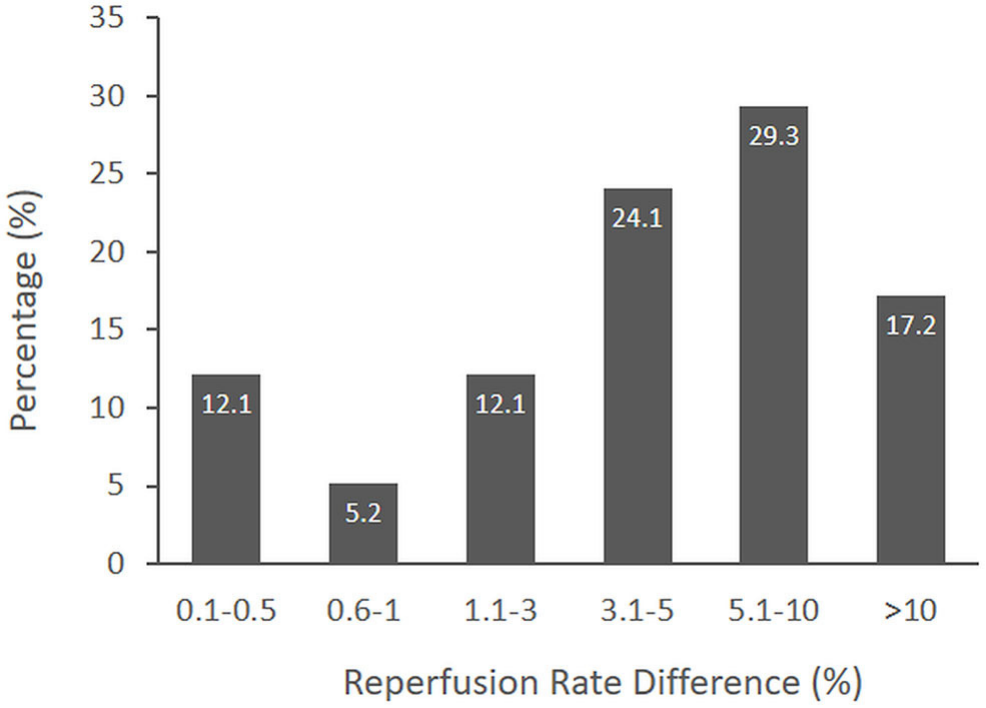
The distribution of MCID responses from surveyed international experts. The median MCID of a novel thrombectomy device to achieve substantial reperfusion was 3.1–5% (IQR 1.1–3% to 5.1–10%).

Figure 1 shows the distribution of responder identifications of the MCID for the substantial reperfusion outcome. The median MCID was 31–50 per 1,000 treated patients (interquartile range, IQR, 11–30 to 51–100). Converted to natural base-100 values, the MCID was 3.1–5% (IQR 1.1–3% to 5.1–10%). Fewer than one fifth of responders considered the MCID to be more than 10%. Early and later responder MCID estimates did not statistically differ. There was a trend for early responders to select higher MCID values than later responders (*p* = 0.07); however, for both groups, the median MCID was 3.1–5%.

**Figure 2:**
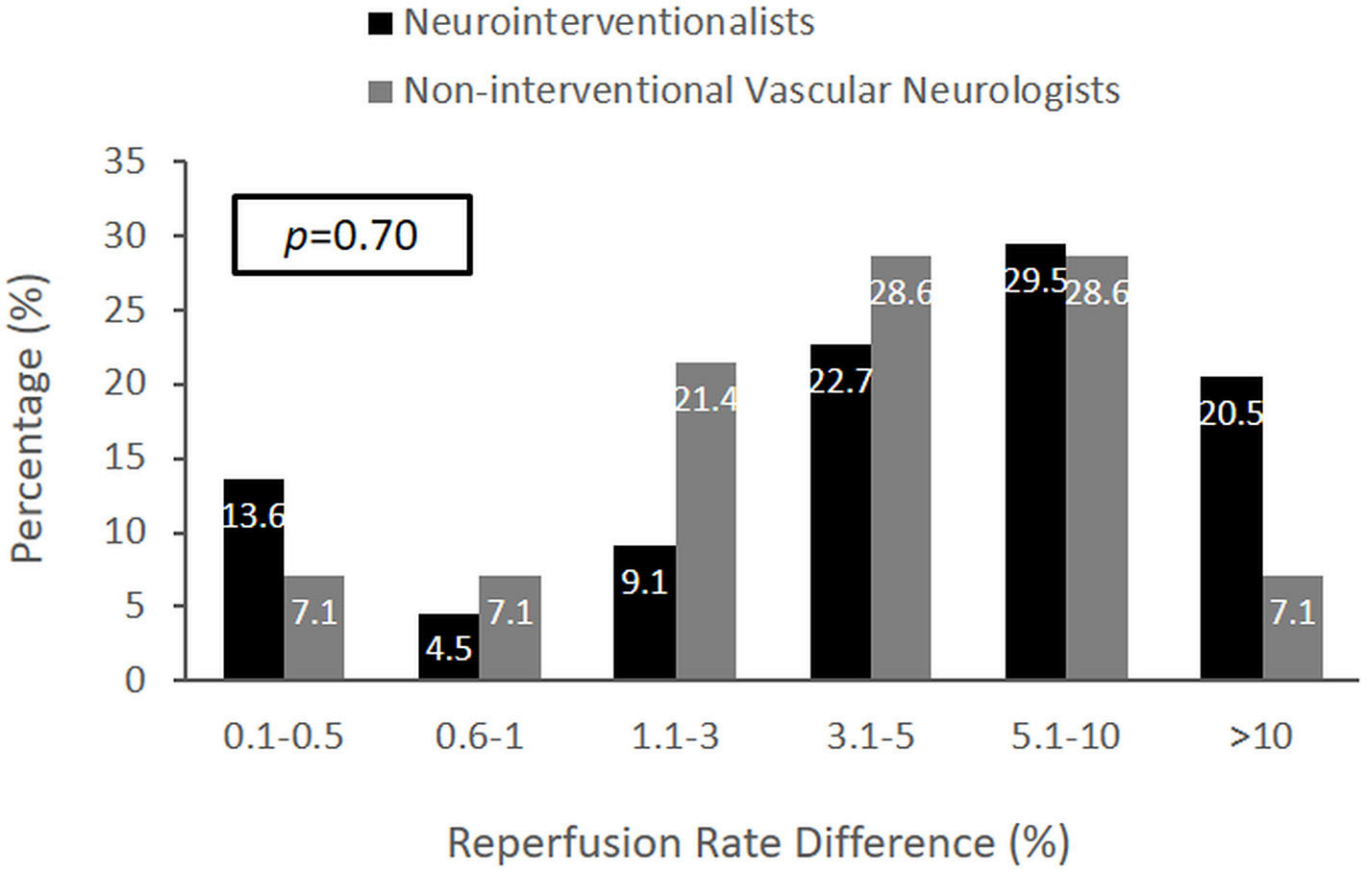
The distribution of MCID responses comparing neuro-interventionalists and noninterventional vascular neurologists. The MCID distributions were not significantly different (*p* = 0.70). However, the median MCID of vascular neurologists was within 3.1–5% (IQR 1.1–3% to 5.1–10%), and the median MCID of neuro-interventionalists was exactly 5% (IQR 1.1–3% to 5.1–10%).

The judgments of neuro-interventionalists compared with noninterventional vascular neurologists regarding the MCID for substantial reperfusion did not show a significant difference (Figure 2). In an analysis by respondent specialty, the median MCID of endovascular neurosurgeons was nominally higher than for other groups (5.1–10% vs. 3.1–5%), but the sample size of surgical specialists was small (*n* = 6), and differences did not reach statistical significance.

## Discussion

This study finds that expert neuro-interventionalists and noninterventional vascular neurologists judge that the MCID for the outcome of substantial reperfusion (TICI 2b-3) within 3 passes for endovascular thrombectomy devices in patients with acute ischemic stroke due to large vessel occlusion is 3.1–5%. The responding neurovascular experts had extensive practice experience, had substantial representation of both interventionalists and noninterventionalists and each of the neuroscience practice specialties, and substantial participation from both North America and Europe although with under-representation of Asia and other regions. Formal responder–nonresponder and continuum-of-resistance tests indicate that the responding experts are representative of all physicians at highly experienced thrombectomy hospital sites.

The findings of this study contrast with the MCID values for a reperfusion technical efficacy primary outcome actually employed in the design of prior endovascular thrombectomy device trials. At least four trials have explicitly incorporated a reperfusion outcome MCID into study design, power calculations, and interpretation. All were noninferiority trials and used MCID values of 8–15%, all substantially higher than the expert-determined 3.1–5% value identified in the current study (10, 11, 19, 20). None of the trial reports indicate how its more generous MCID values were derived. Though the difference in performance of the devices actually fell within the range of MCID value derived from the current expert survey for several trials (11, 19, 20), a larger MCID allows noninferiority trials to use smaller sample sizes and still have adequate power to narrow confidence intervals to fall within the selected MCID. However, if a selected MCID is higher than the true MCID for an outcome, the trial's declaration of success in demonstrating noninferiority may not be valid.

Given the discrepancy between the formal expert survey–derived MCID value in the current study and MCID values actually used in prior trials, it is helpful to consider other sources of perspective on the MCID. Two important additional frames for considering MCID values are (1) stroke patient clinical functional outcomes and (2) MCID values used for similar technical efficacy outcomes in other disease states.

With regard to stroke patient clinical outcomes, a recent meta-analysis, including the five major randomized controlled trials of stent retrievers, analyzed the relationship between increased achievement of substantial reperfusion and increased attainment of excellent (modified Rankin Scale, mRS, 0–1) and good (mRS 0–2) global disability outcomes at 3 months poststroke. The analysis showed that every 10% absolute increase in the rate of substantial reperfusion within the range of 60–80% was associated with a 17% absolute increase in excellent outcome (mRS 0–1) and an 11% absolute increase in good outcome (mRS 0–2) (21). Prior trials and expert-survey studies have identified three potential MCID values for dichotomized 3-month global disability outcomes: 6.5% (22), 5% (23), and 1.3% (16). Based on the strength of the association of increased substantial reperfusion with increased favorable 3-month disability outcomes, these MCIDs for final functional outcome are equivalent to MCIDs for substantial reperfusion of 3.8% 2.9, and 0.8% (based on excellent mRS 0–1 outcome) and 5.9, 4.2, and 1.2% (based on good mRS 0–2 outcome). The higher range of these values (2.9–5.9%) are convergent with the expert-derived MCID value (3.1–5% for substantial reperfusion) in the current study and also well below the reperfusion MCID values actually employed in prior trials.

With regard to MCID values used for similar efficacy outcomes in other disease states, cardiac trials of novel coronary artery stent devices to treat acute myocardial infarction are of relevance. The framework for coronary revascularization trial endpoints differs from cerebrovascular trials because successful initial recanalizaton rates with primary stents of all types are extremely high: over 98%. The important performance feature potentially differentiating cardiac stents is durability of recanalization. Recent trials have, therefore, used a primary outcome of 12-month target lesion failure, defined as the composite occurrence of any ischemia-driven revascularization of the target lesion, myocardial infarction related to the target vessel, or any cardiac death. For this endpoint, contemporary cardiac stent trials have used noninferiority margins of 3.5–4.4% (24–26). Accordingly, the MCID values used in cardiac reperfusion trials are within the same ranges as the MCID value for cerebral reperfusion trials determined by neurovascular experts in in the current study.

The wording of the case scenario presented to the surveyed experts purposely did not specify if the patient was an alteplase-failure or alteplase-ineligible patient. Because shorter surveys are more often fully completed, making question compression is desirable if potential scenarios are sufficiently similar. The survey used a single, prototypical MCID case-eliciting scenario that covered both possibilities. Available evidence indicates that the MCID value for achievement of substantial reperfusion is similar for (a) an LVO-AIS patient who has not spontaneously recanalized and is not eligible for alteplase and (b) an LVO-AIS patient who has not spontaneously recanalized and also has not recanalized with alteplase. In both cases, the clinical benefit of achievement within the next 20–60 min of substantial reperfusion with EVT has similar (and substantial) value.

Survey questionnaires may have anchoring, centrality, and imprecision bias. Anchoring bias is the tendency of individuals to rely too heavily, or “anchor,” on the first trait or piece of information when making decisions (27). Centrality bias is the tendency of test takers to select responses placed in middle positions in multiple-choice questions (28, 29). Imprecision bias arises when survey response options each have overly broad numeric ranges. We designed the questionnaire to minimize these biases. With regard to framing, the survey text avoided providing anchoring information regarding MCIDs used in prior trials or suggested by individual experts. With regard to centrality, the survey provided an even number (6) of response options, eliminating a default pure middle choice (30). In addition, the survey provided a larger number of response options (6 rather than 2–5) to increase precision. The distribution of responses showed no obvious tendency toward centralization.

There are limitations to this study. First, 56% of survey recipients and 62% of responders were from North America. The list was derived from contemporary trials relevant to thrombectomy device development. These experts had appropriate experience to knowledgeably make assessments of MCIDs for device performance. With the diffusion of endovascular thrombectomy worldwide, more and more individuals are accruing this expertise. Replication with experts drawn more extensively from other regions is desirable to demonstrate generalizability. Second, the survey response rate was only moderate. However, comparing demographic characteristics of responders and nonresponders showed no group differences and continuum of resistance analysis similarly indicated absence of responder bias. Third, the MCID case-eliciting scenario used in this expert survey envisioned a patient with an M1 MCA occlusion. It is possible that experts' MCID values may differ modestly for different LVO target locations; the MCID for the intracranial internal carotid artery may be slightly different from that for the M1 MCA, which, in turn, might slightly differ from that for the basilar artery. Future artery-specific MCID characterization studies are desirable. Fourth, the technical efficacy outcome of substantial reperfusion within 3 passes, although it is the leading technical endpoint used in recent trials, is beginning to reach a ceiling effect with achievement rates approaching 85–90% in recent trials. More stringent reperfusion endpoints, such as first pass excellent reperfusion (FP-TICI 2c-3) or time to achievement of substantial TICI 2b-3 reperfusion are associated with further incremental gains in final clinical outcome and may play more important roles as technical efficacy outcomes in clinical trials in the future (31). The MCID for FP-TICI 2c-3 and other, more advanced, final reperfusion status outcomes are likely to be similar but not exactly equal to that for substantial reperfusion within 3 passes.

## Conclusions

This study indicates that neurovascular experts judge the minimal clinically important difference for endovascular thrombectomy devices in achieving substantial reperfusion is 3.1–5%. This MCID, which is lower than the noninferiority margins used in several recent clinical trials comparing novel and standard thrombectomy devices, can inform clinical trial design and device development.

## Data Availability Statement

All datasets generated for this study are included in the article/supplementary material.

## Ethics Statement

Ethical review and approval was not required for the study on human participants in accordance with the local legislation and institutional requirements. Written informed consent from the participants was not required to participate in this study in accordance with the national legislation and the institutional requirements.

## Author Contributions

JS and C-JL: conception or design and reviewed submitted version. C-JL: acquisition, analysis, or interpretation of data and manuscript drafting. N/A: technical support. JS: critical revision for important intellectual content and final approval of the version to be published. All authors contributed to the article and approved the submitted version.

## Conflict of Interest

JS is an employee of the University of California. The University of California has patent rights in retrieval devices for stroke. JS has served as an unpaid site investigator in multicenter trials sponsored by Medtronic, Stryker, and Neuravia, for which the UC Regents received payments on the basis of clinical trial contracts for the number of subjects enrolled; has received funding for services as a scientific consultant regarding trial design and conduct to Medtronic, Stryker, Neuravi/Cerenovus, and Boehringer Ingelheim (prevention only), and stock options for services as a scientific consultant regarding trial design and conduct to Rapid Medical. The remaining author declares that the research was conducted in the absence of any commercial or financial relationships that could be construed as a potential conflict of interest.

## Abbreviations

AIS-LVO: acute ischemic stroke due to large vessel occlusion
EVT: endovascular thrombectomy
MCID: minimal clinically important difference
mRS: modified rankin scale
TICI: thrombolysis in cerebral infarction.
